# Inhibition of circular RNA CDR1as increases chemosensitivity of 5‐FU‐resistant BC cells through up‐regulating miR‐7

**DOI:** 10.1111/jcmm.14171

**Published:** 2019-03-18

**Authors:** Wei Yang, Juan Gu, Xuedong Wang, Yueping Wang, Mei Feng, Daoping Zhou, Jianmin Guo, Ming Zhou

**Affiliations:** ^1^ Guangdong Key Laboratory of Animal Conservation and Resource Utilization, Guangdong Public Laboratory Wild Animal Conservation and Utilization Guangdong Institute of Applied Biological Resources Guangzhou China; ^2^ Department of Medical Laboratory Science The Fifth People’s Hospital of Wuxi, The Medical School of Jiangnan University Wuxi Jiangsu China; ^3^ Department of Pathology The Fifth People’s Hospital of Wuxi, Nanjing Medical University Wuxi Jiangsu China; ^4^ Department of Medical Laboratory Science The Second People’s Hospital of Anhui Province, Anhui Medical University Hefei Anhui China; ^5^ Department of Biology, College of Arts & Science Massachusetts University Boston Massachusetts; ^6^ Guangdong Lewwin Pharmaceutical Research Institute Co.,Ltd Guangzhou Guangdong China; ^7^ Cancer Research Institute, Central South University Changsha Hunan China

**Keywords:** 5‐Fu, breast cancer, CCNE1, CDR1as, circular RNA, drug resistance, miR‐7

## Abstract

This study aims to explore the mechanism of Circular RNA CDR1as implicating in regulating 5‐fluorouracil (5‐FU) chemosensitivity in breast cancer (BC) by competitively inhibiting miR‐7 to regulate CCNE1. Expressions of CDR1as and miR‐7 in 5‐FU‐resistant BC cells were determined by RT‐PCR. CCK‐8, colony formation assay and flow cytometry were applied to measure half maximal inhibitory concentration (IC50), 5‐Fu chemosensitivity and cell apoptosis. Western blot was used to detect the expressions of apoptosis‐related factors. CDR1as was elevated while miR‐7 was inhibited in 5‐FU‐resistant BC cells. Cells transfected with si‐CDR1as or miR‐7 mimic had decreased IC50 and colony formation rate, increased expressions of Bax/Bcl2 and cleaved‐Caspase‐3/Caspase‐3, indicating inhibition of CDR1as and overexpression of miR‐7 enhances the chemosensitity of 5‐FU‐resistant BC cells. Targetscan software indicates a binding site of CDR1as and miR‐7 and that CCNE1 is a target gene of miR‐7. miR‐7 can gather CDR1as in BC cells and can inhibit CCNE1. In comparison to si‐CDR1as group, CCNE1 was increased and chemosensitivity to 5‐Fu was suppressed in si‐CDR1as + miR‐7 inhibitor group. When compared with miR‐7 mimic group, CDR1as + miR‐7 mimic group had increased CCNE1 and decreased chemosensitivity to 5‐Fu. Nude mouse model of BC demonstrated that the growth of xenotransplanted tumour in si‐CDR1as + miR‐7 inhibitor group was faster than that in si‐CDR1as group. The tumour growth in CDR1as + miR‐7 mimic group was faster than that in miR‐7 mimic group. CDR1as may regulate chemosensitivity of 5‐FU‐resistant BC cells by inhibiting miR‐7 to regulate CCNE1.

## 1 INTRODUCTION

Breast cancer (BC) is one of the most prevalent and death related cancer in female globally and is a major threat to public health with incidence only secondary to lung cancer.^1^, ^2^ Most of BC cases are found in women with age more than 50 years old while nowadays witnesses an increasing trend on incidence happened in younger and women aged 20~34 years and 35~44 years with a respective incidence of 1.9% and 10.5%.^3^ Although improvements in early detection and systemic therapy have significantly decreased recurrence and prolonged survival, data reported that 30% of the women diagnosed with early‐stage BC in turn progress to metastatic BC, for which therapeutic options are limited.^4^, ^5^ With prolonged survival and tumour recurrence, serious problems emerge, such as accumulated drug dosages that approach the upper limit of safety, therapy‐related toxicity and drug resistance.^6^ Consequently, there is an ever‐increasing need for new drugs or combination regimens for the treatment of BC.

5‐fluorouracil (5‐FU) is well‐known for its anti‐tumour effect in numerous tumour, including BC, colon cancer and some skin cancers, which can be paired with other drugs based on individual conditions, cancer types and expected outcomes.^7^ The side effect of 5‐FU includes inflammation of the mouth, loss of appetite, low blood cell counts, hair loss and skin inflammation.^8^ Generally, 5‐FU is commonly used to treat BC. Nevertheless, it becomes increasingly ineffective with tumour progression due to chemoresistance.^9^

Circular RNA (CircRNA) is a naturally occurring family of noncoding RNAs, which has been commonly detected in viruses, plant and animals.^10^, ^11^, ^12^ CircRNAs are proved as potential biomarkers in many diseases including hepatoma carcinoma and other cancers.^13^ CDR1as (also known as CiRS‐7), one of the thousands of CircRNAs, was recently demonstrated to function as a powerful miR‐7 sponge/inhibitor in developing midbrain of zebrafish, suggesting a novel mechanism for regulating microRNA functions.^14^ Evidence supported that ectopic expression of CDR1as induced midbrain brain defects, which was similar with the phenotypes found in the knockdown of miR‐7.^15^ Although previous study has focused emphasis on the relationship between CDR1as and miR‐7, little study was published whether CDR1as can regulate the chemosensitivity to 5‐FU in BC cells through targeting miR‐7. This study was conducted to investigate the mechanism of CDR1as implicating in regulating 5‐FU chemosensitivity in BC by competitively inhibiting miR‐7 to regulate CCNE1.

## 2 MATERIALS AND METHODS

### 2.1 Cell cultivation

BC cell lines (MCF‐7, SKBR‐3, MDA‐MB‐231 and HCC‐1937) and normal mammary epithelial cells (MCF10) were purchased from cell bank of Chinese Academy of Science. The 5‐FU‐resistant BC cells (MCF‐7/5‐Fu, SKBR‐3/5‐Fu, MDA‐MB‐231/5‐Fu and HCC‐1937/5‐Fu) were obtained in this lab using concentration gradient method. BC cells were incubated in RPMI1640 complete culture medium containing 10% FBS in an incubator at 37°C with 5% CO_2_. Once cell proliferation density reaches approximately 90%, 5‐Fu (purchased from Sigma, Saint Louis, MO, USA) was added till the concentration of 5‐Fu reaches 3.84 µmol/L. Then cells were cultivated for another 48 hours before sub‐cultivation using new culture medium. Gradually increase the concentration of 5‐Fu till it reaches 23.0 µmol/L^16^ to obtain MCF‐7/5‐Fu, SKBR‐3/5‐Fu, MDA‐MB‐231/5‐Fu and HCC‐1937/5‐Fu cells.

### 2.2 Cell grouping

Further experiments were conducted on MCF‐7 cells and MDA‐MB‐231 cells, the former has the largest difference with MCF10 CDR1as cells and the latter has the least difference with MCF10 CDR1as cells. The two kinds of cells were induced for drug resistance and grouped into following groups: Blank group, Empty plasmid group, si‐CDR1as group, CDR1as group, negative control (NC) group, miR‐7 mimic group, miR‐7 inhibitor group, si‐CDR1as + miR‐7 inhibitor and CDR1as + miR‐7 mimic group. No treatment on blank group, Empty plasmid group transfecting with empty plasma, si‐CDR1as group transfecting with siRNA interference plasmid of CDR1as, CDR1as group transfecting with plasma with overexpression of CDR1as, NC group transfecting with NC sequence of miR‐7, miR‐7 mimic group transfecting with mimics of miR‐7, miR‐7a inhibitor group transfecting with inhibitor of miR‐7, si‐CDR1as+ miR‐7 inhibitor group transfecting with siRNA interference plasmid of CDR1as and inhibitor of miR‐7, CDR1as + miR‐7 mimic group transfecting with plasma with overexpression of CDR1as and mimics of miR‐7a. Empty plasma, siRNA interference plasmid, overexpression plasma as well as NC, mimics and inhibitor of miR‐7 were all purchased from Shanghai GenePharma Co., Ltd. Cell transfection was conducted based on the introduction of Lipofectamine 2000 (Invitrogen, Carlsbad, CA, USA). Cells in each group were incubated in incubators for 48 h before further experiments.

### 2.3 Reverse transcript polymerase chain reaction (RT‐PCR)

The TRIzol reagent (Invitrogen, Carlsbad, CA, USA) was used to isolate total RNA, including microRNA, according to the manufacturer's instructions. One microgram of total mRNA was used to reverse transcribe cDNA with Takara RT‐PCR synthesis kit, according to the manufacturer's instructions (TAKARA, Dalian, China). The cDNA was performed using SYBR Premix Ex Taq II (TAKARA, Dalian, China) on the PikoReal 96 qPCR system (Thermo Scientific, United States). The mRNA level of β‐actin was relative to internal control. For miR‐7a expression, microRNA was analyzed by using the TaqMan MicroRNA Reverse Transcription Kit (Applied Biosystems, Foster City, CA, USA) with provided RT‐U6 and microRNA‐specific stem‐loop primers, and the expression levels were determined through TaqMan MicroRNA assays with the TaqMan Universal PCR Master Mix (Applied Biosystems), and U6 snRNA was used as the endogenous control. All reactions were preceded on the ABI 7500 real‐time PCR system (Applied Biosystems) by standard protocols. The sequences used for PCR is listed in Table 1. Data were analyzed using 2^−ΔΔCt^ method. The experiments were conducted for three times to obtain average value.

**Table 1:** Primer sequences for reverse transcript polymerase chain reaction

Gene	Sequences
CDR1as	5′‐ACGTCTCCAGTGTGCTGA‐3′
	5′‐CTTGACACAGGTGCCATC‐3′
β‐actin	5′‐GTCACCCACACTGTGCCCATC‐3′
	5′‐ACAGAGTACTTGCGCTCAGGA‐3′
miR‐7	5′‐TGGAAGACTAGTGATTTTGTTGT‐3′
U6	5′‐TGGAAGACTAGTGATTTTGTTGT‐3′

### 2.4 CCK‐8

Cells in each group were digested and inoculated in 96‐well plate at the density of 8 × 10^3^/well. The volume of each well was 200 uL. When cells were adherent to the wall of each well, 5‐Fu of respective concentration of 0, 0.10 umol/L, 0.50 umol/L, 2.50 umol/L, 12.50 umol/L, 20.00 umol/L and 40.00 umol/L were added. Three duplicate wells were set for each concentration. Blank well and control group were also established. The plate was incubated at 37°C with 5% CO_2 _for 48 hours before CCK‐8 assay was applied. Replace the culture medium with 100 uL of fresh culture medium which contains 10 uL of CCK‐8 regent (Beyotime Biotechnology Co., Ltd.). Then the plate was maintained in an incubator for 2 hours before the microplate reader (Bio‐Rad, USA) was utilized to measure the optical density (OD) at the wavelength of 450 nm. Cell survival rate was calculated and cell growth curve was accordingly drawn. The experiment was repeated for three times. Half maximal inhibitory concentration (IC50) was calculated using Probit regression analysis by SPSS software.

### 2.5 Colony formation assay

Cells in logarithmic phase were digested by pancreatic enzymes and made into single cell suspension. About 200 cells were separately inoculated into a 6 cm dish and incubated in complete culture medium containing 15 nmol/mL of 5‐Fu. Then the dishes were cultured in an incubator at 37°C with 5% CO_2 _for 2‐3 weeks, during which the culture medium will not be replaced. Terminate the cultivation and abandon the culture medium until cell cloning was visible by naked eyes. The dishes were washed in PBS for twice and then added 5 mL of methanol solution for fixation at room temperature for 15 minutes. After that, the fixation solution was absorbed by using a vacuum pump and Giemsa dye (SIGMA, USA) was used for staining. About 30 minutes later, the staining solution was gradually washed away and the dishes were dried in the air. With the application of an inverted microscope, cell colon number and colon formation rate were calculated by naked eyes (colon formation rate = cell colon number/number of inoculated cells × 100%).

### 2.6 Flow cytometry (FCM)

AnnexinV/propidiom iodide (PI) double staining method was applied to detect cell apoptosis. Cells after grouping for 48 hours were collected for concentration adjustment to 1 × 10^6^/mL. Then 0.5 mL of cell suspension was added into a centrifuge tube which was then added 1.25 uL of AnnexinV‐FITC (NanJing KeyGen Biotech Co., Ltd.) for reaction without light exposure at room temperature for 15 minutes. Later, the centrifuge tube was subjected to centrifugation at 1000 rpm for 5 minutes. Abandon the supernatant and re‐suspend the cells with 0.5 mL of pre‐cold binding buffer. FCM instrument (BD, USA) was utilized for cell apoptosis immediately after 10 uL of PI was added. Cells in the lower left chamber (Q4) of the scatter diagram were healthy cells (FITC−/PI−), cells in lower right chamber (Q3) were early apoptotic cells (FITC+/PI−), cells in upper right chamber (Q2) were necrotic cells and advanced apoptotic cells (FITC+/PI+). Apoptosis rate = percentage of early apoptosis (Q3) + percentage of advanced apoptosis (Q2).

### 2.7 Western blot analysis

Total protein of cells in each group was isolated using protein lysis buffer and Bradford method (Thermo Fisher Scientific, Waltham, MA, USA) was applied for protein quantitation. Protein of 50 μg was firstly subjected to sodium dodecyl sulfate polyacrylamide gel electrophoresis (SDS‐PAGE) and then transferred to Polyvinylidene Fluoride membrane (Millipore, Billerica, MA, USA). Then 5% skimmed milk power was added at 37°C to terminate the reaction for 1 hour. After that, primary antibodies of mouse anti human CNE1 (ab3927, 1:1000), Bcl2 (ab32124, 1:1000), Bax (ab32503, 1:1000), Caspase3 (ab32503, 1:1000), Cleaved‐Caspase3 (ab32042, 1:100), β‐actin (1:1000, Abcam) (all purchased from Abcam, Cambridge, MA, USA) were added at 4°C for overnight. The membrane was washed with PBST for three times, each for 5 minutes before secondary antibodies of rabbit anti mouse which were labeled by horse radish peroxidase (1:2000, Abcam, Cambridge, MA, USA) were added for incubation at room temperature for 2 hours. Wash the membrane again. The images were observed after ECL solution (Amersham Bioscience, Uppsala, Sweden) was added. Scion Image analysis software (Scion Corporation, Frederick, MD, USA) was used to analyze the protein brand. The relative protein expression shall be the ratio of OD of target protein and β‐actin.

### 2.8 RNA pull down assay

The biotinylated miR‐7 and mutant miR‐7 (miR‐7‐MUT) which were designed and synthesized by Invitrogen and transfected into MCF‐7 cells and 48 hours later, RNA pull down lysate was applied to lyse cells. Take 100 ug samples as input for further experiments. Streptavidin magnetic beads (S1421S, NEB, Ipswich, MA, USA) was added and incubated at 4°C for 3 hours. Abandon the supernatant. Samples were added with Trizol to isolated RNA which was bind to the beads. PCR amplification was performed to detect the expression of CDR1as in target genes.

### 2.9 Double luciferase reporter assay

Using Targetscan database to evaluate the target genes of miR‐7, which preliminary determined CCNE1 as a direct target gene of miR‐7 for further experiments. Double luciferase reporter assay was applied to verify whether miR‐7 can inhibit CCNE1. The overall length of 3′UTR of CCNE1 amplified gene was cloned to the multiple cloning sites on the downstream of pmirGLO (Promega) Luciferase. Target gene database was used to predict the bind site of miR‐7 and its target gene. Then the bind site of the target gene was mutant (CCNE1‐Mut). Expression vector of ranilla luciferase, pRL‐TK (TaKaRa) was used as internal control. miR‐7 mimics and expression vector of luciferase reporter (CCNE1‐Wt and CCNE1‐Mut) as well as NC sequence and expression vector of luciferase reporter (CCNE1‐Wt and CCNE1‐Mut) were separately co‐transfected into MCF‐7 cells. The activity of double luciferase was measured according to the method proposed by Promega for three times.

### 2.10 Xenotransplanted tumour in nude mouse

Animal experiments were conducted in strict accordance with the approved animal protocols and guidelines established by Medicine Ethics Review Committee for animal experiments of The Fifth People's Hospital of Wuxi. BALB/C nude female mice (N = 120) aged 6 weeks which were purchased from animal center of Peking Union Medical College Hospital were weighted from 16 g to 21 g. All mice were fed in laminar flow cabinet in specific pathogen free condition with constant temperature and humidity. The cabinet shall be disinfected at a regular basis. Padding, water and fodder were changed a regular and sterile basis. About 0.2 mL of suspended solution (5 × 10^6^ MCF‐7/5‐Fu cells at logarithmic phase) was separately subjected to subcutaneous injection in the axilla of anterior limb of each nude mouse (N = 120). Two weeks later, nodes of size of 100 mm^3 ^were observed in mice. Then the 120 mice were randomly grouped into Empty plasmid group (injected with empty plasma), si‐CDR1as group (injected with siRNA of CDR1as), CDR1as group (injected with plasma with overexpression of CDR1as), miR‐7 mimic group (injected with miR‐7a agomirs, a modified miRNA which was more stable than miRNA mimic), miR‐7 inhibitor group (injected with anti‐miR‐7a antagomirs), si‐CDR1as+ miR‐7 inhibitor group (injected with siRNA of CDR1as and anti‐miR‐7a antagomirs) and CDR1as + miR‐7 mimic group (injected with plasma with overexpression of CDR1as and miR‐7a agomirs), each group had 20 mice. The injection was performed once a week with concentration of 30 μg/200 uL/time, total four injections were need. Meanwhile, mice in each group were received intravenous injection of 5‐Fu (10 mg/kg) through the tails. The tumour size and reaction of each mouse were observed and recorded. The injection shall be continuous for 3 days. The long (a) and short (b) diameter of the tumour were measured and the tumour growth curve were calculated according to the formula *V* = *ab* ^2^/2. After the injection was finished, the tumour samples shall be collected in sterile condition and be subjected to pathological slice and frozen at −80°C for further usage.

### 2.11 Statistical analysis

SPSS 22.0 software (SPSS, Inc, Chicago, IL, USA) was utilized for data analysis. Measurement data were expressed as mean ±standard deviation. Pairwise comparison was conducted using least significant difference method. Comparisons among groups were analyzed by one‐way ANOVA. Data complying with normal distribution were compared by *t* test. *P* < 0.05 was considered as significant difference.

## 3 RESULTS

### 3.1 Inhibition of CDR1as increases chemosensitivity of 5‐FU‐resistant BC cells

Compared with MCF10A cells, the BC cells (MCF‐7, SKBR‐3, MDA‐MB‐231 and HCC‐1937) had substantially increased CDR1as expression, among which MCF‐7 cells had the highest CDR1as expression and MDA‐MB‐23 cells had the lowest CDR1as expression, therefore, both MCF‐7 cells and MDA‐MB‐23 cells were selected for further experiments.

**Figure 1:**
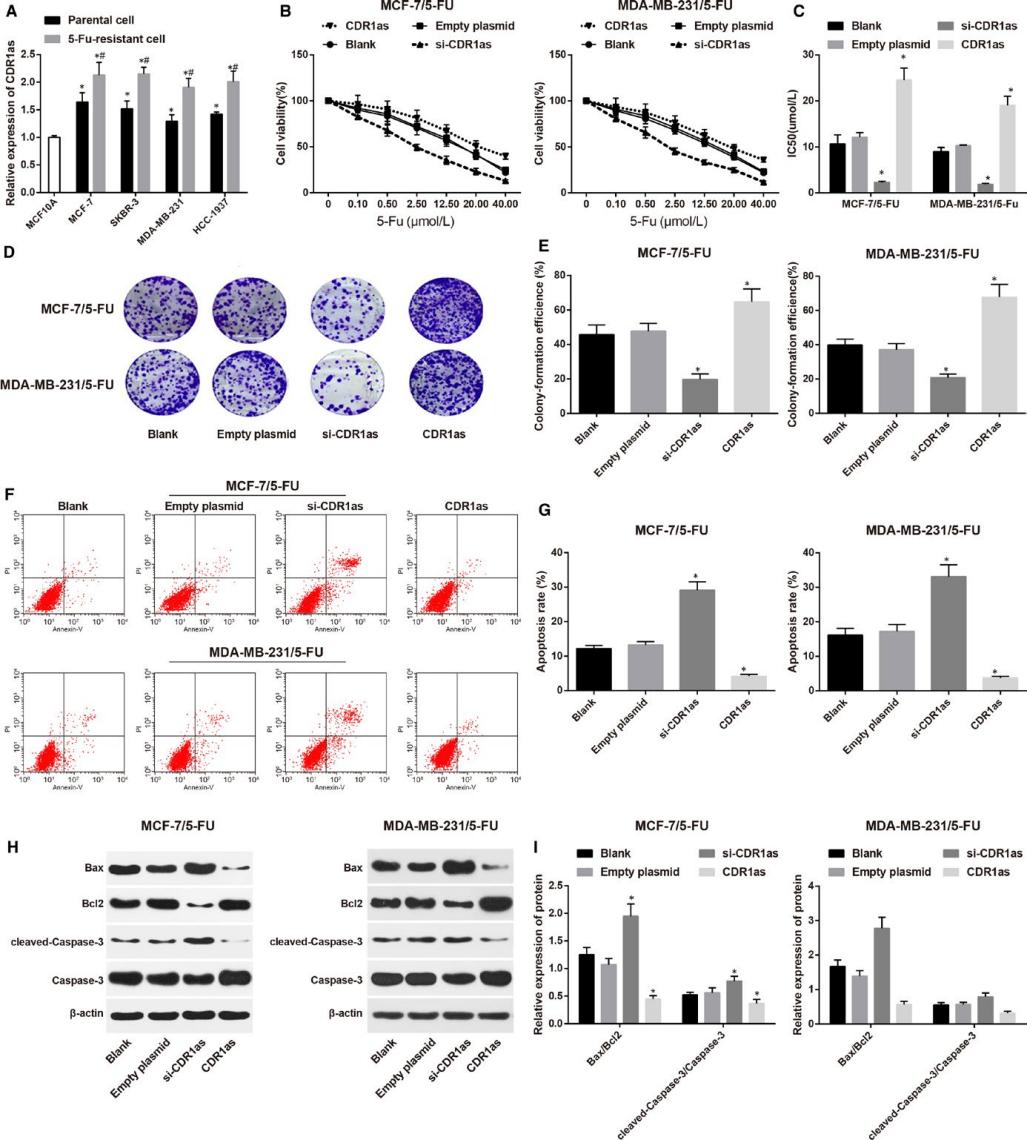
Effect of overexpression or suppression of CDR1as on chemosensitivity of 5‐fluorouracil (5‐FU)‐resistant BC cells. (A) Expressions of CDR1as in BC cells and their corresponding 5‐FU‐resistant BC cells; (B), cell growth curve of 5‐FU‐resistant BC cells in each group after treatment by different concentration of 5‐Fu; (C), IC50 of 5‐FU‐resistant BC cells in each group; (D), colon formation images of 5‐FU‐resistant BC cells in each group by colon formation assay; (E), colon formation rate of 5‐FU‐resistant BC cells in each group; (F), cell apoptosis of 5‐FU‐resistant BC cells in each group; (G), cell apoptosis rate of 5‐FU‐resistant BC cells in each group; (H), Western blot on apoptosis related factors of 5‐FU‐resistant BC cells in each group; (I), expressions of apoptosis related factors of 5‐FU‐resistant BC cells in each group; ^*^, compared with Blank group, *P < *0.05; BC, breast cancer; IC50, half maximal inhibitory concentration

Compared with BC cells (MCF‐7, SKBR‐3, MDA‐MB‐231 and HCC‐1937), the corresponding 5‐Fu‐resistant BC cells (MCF‐7/5‐Fu, SKBR‐3/5‐Fu, MDA‐MB‐231/5‐Fu and HCC‐1937/5‐Fu) had elevated CDR1as expression (all *P < *0.05) (Figure 1A), indicating that CDR1as may have certain effect on the chemosensitivity of BC cells to 5‐Fu.

MCF‐7/5‐Fu cells and MDA‐MB‐231/5‐Fu cells were separately transfected with si‐CDR1as sequence and CDR1as sequence, followed by treatment of 5‐Fu in different concentration. CCK‐8 was applied to measure the cell proliferation. The cell survival rate of both MCF‐7/5‐Fu and MDA‐MB‐231/5‐Fu cells were decreased along with the increased concentration of 5‐Fu (Figure 1B). Analysis on IC50 showed no significant difference between the Blank group and Empty plasmid group both in MCF‐7/5‐Fu cells and MDA‐MB‐231/5‐Fu cells (both *P* > 0.05). Interestingly, in comparison to MCF‐7/5‐Fu cells and MDA‐MB‐231/5‐Fu cells in Empty plasmid group, the IC50 in si‐CDR1as group was substantially decreased while that in CDR1as group was elevated (both *P < *0.05) (Figure 1C). Colony formation assay demonstrated that the colon formation rat of both MCF‐7/5‐Fu cells and MDA‐MB‐231/5‐Fu cells in Blank group was not different from that in Empty plasmid group (both *P* > 0.05). In contrast to Empty plasmid group, the colon formation rate of both MCF‐7/5‐Fu cells and MDA‐MB‐231/5‐Fu cells in si‐CDR1as was suppressed, while that of CDR1as group was increased (all *P < *0.05) (Figure 1D,E).

Detection on cell apoptosis (Figure 1F,G) showed no significant difference on both MCF‐7/5‐Fu cells and MDA‐MB‐231/5‐Fu cells between Blank group and Empty plasmid group (both *P* > 0.05). The cell apoptosis rate in si‐CDR1as group was higher than that in Empty plasmid group, while that in CDR1as group was lower than that in Empty plasmid group (all *P < *0.05). Measurement on apoptosis related factors is illustrated in Figure 1H,I. In both MCF‐7/5‐Fu cells and MDA‐MB‐231/5‐Fu cells, the expressions of Bax/Bcl2 and cleaved‐Caspase‐3/Caspase‐3 in si‐CDR1a group were increased, while those in CDR1as group were suppressed when compared to those in Empty plasmid group, suggesting that suppression on CDR1as may increase chemosensitivity of 5‐FU‐resistant BC cells.

### 3.2 Overexpression of miR‐7 may increase chemosensitivity of 5‐FU‐resistant BC cells

**Figure 2:**
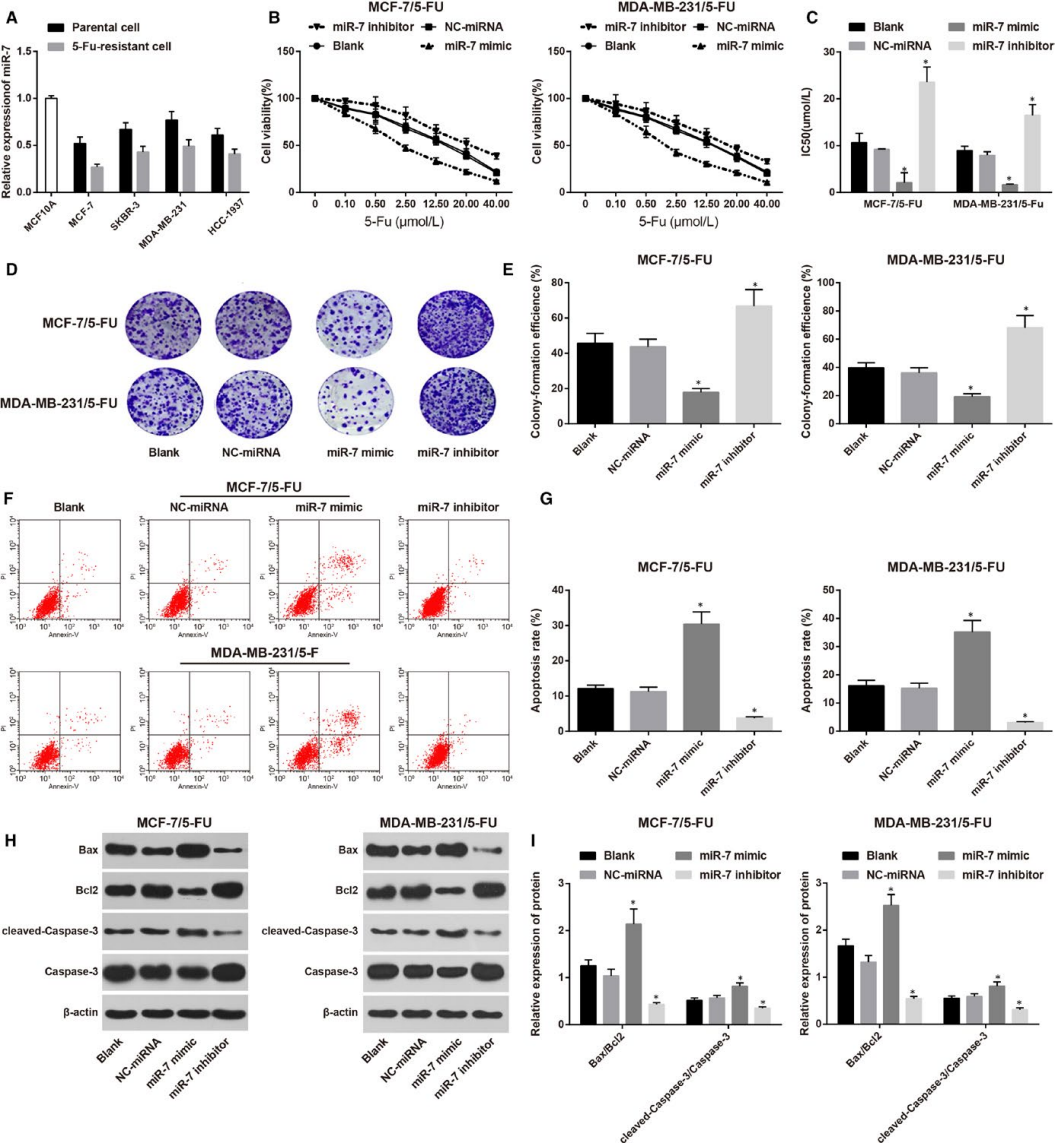
Effect of overexpression or suppression of miR‐7 on chemosensitivity of 5‐fluorouracil (5‐FU)‐resistant BC cells. (A) Expressions of miR‐7 in BC cells; (B), cell growth curve of 5‐FU‐resistant BC cells after being transfected with miR‐7 mimic or miR‐7 inhibitor; (C), IC50 of 5‐FU‐resistant BC cells after being transfected with miR‐7 mimic or miR‐7 inhibitor in each group; (D), colon formation images of 5‐FU‐resistant BC cells after being transfected with miR‐7 mimic or miR‐7 inhibitor; (E), colon formation rate of 5‐FU‐resistant BC cells after being transfected with miR‐7 mimic or miR‐7 inhibitor in each group; (F), cell apoptosis of 5‐FU‐resistant BC cells after being transfected with miR‐7 mimic or miR‐7 inhibitor by FCM; (G), cell apoptosis rate of 5‐FU‐resistant BC cells after being transfected with miR‐7 mimic or miR‐7 inhibitor in each group; (H), western blot on apoptosis related factors of 5‐FU‐resistant BC cells after being transfected with miR‐7 mimic or miR‐7 inhibitor; (I), expressions of apoptosis related factors of 5‐FU‐resistant BC cells after being transfected with miR‐7 mimic or miR‐7 inhibitor; *, compared with Blank group, *P < *0.05; BC, breast cancer; IC50, half maximal inhibitory concentration

Compared with MCF10A cells, BC cells (MCF‐7, SKBR‐3, MDA‐MB‐231 and HCC‐1937) had decreased expression of miR‐7, while in comparison to BC cells, their corresponding 5‐FU‐resistant cells (MCF‐7/5‐Fu, SKBR‐3/5‐Fu, MDA‐MB‐231/5‐Fu and HCC‐1937/5‐Fu) had further suppressed expression of miR‐7 (Figure 2A). miR‐7 mimic and miR‐7 inhibitor were separately transfected into MCF‐7/5‐Fu cells and MDA‐MB‐231/5‐Fu. CCK‐8 was applied to detect cell proliferation rate in each group. The results showed that the cell survival rate of both MCF‐7/5‐Fu cells and MDA‐MB‐231/5‐Fu cells in each group were decreased along with the increased concentration of 5‐Fu (Figure 2B). Comparison on IC50 between NC‐miRNA group and Blank group showed no significant difference. As illustrated in Figure 2C, the IC50 in miR‐7 mimic group was reduced, while that in miR‐7 inhibitor group was increased when compared with NC‐miRNA group. The results of colon formation assay showed (Figure 2D,E) that the colon formation rate in miR‐7 mimic group was lower than that in NC‐miRNA group, while that in miR‐7 inhibitor group was higher than that in NC‐miRNA group. No significant difference was found between NC‐miRNA group and Blank group. Comparison on cell apoptosis rate is demonstrated in Figure 2F,G. In contrast to NC‐miRNA group, the cell apoptosis rate in miR‐7 mimic group was increased while that in miR‐7 inhibitor group was inhibited. Expressions of apoptosis related factors (Figure 2H,I) showed that the expressions of Bax/Bcl2 and cleaved‐Caspase‐3/Caspase‐3 in miR‐7 mimic group were higher than that in NC‐miRNA group, while miR‐7 inhibitor group had suppressed expressions when compared with NC‐miRNA group. Those results implied that overexpression of miR‐7 may increase chemosensitivity of 5‐FU‐resistant BC cells.

### 3.3 CDR1as competitively inhibits miR‐7 to regulate CCNE1

**Figure 3:**
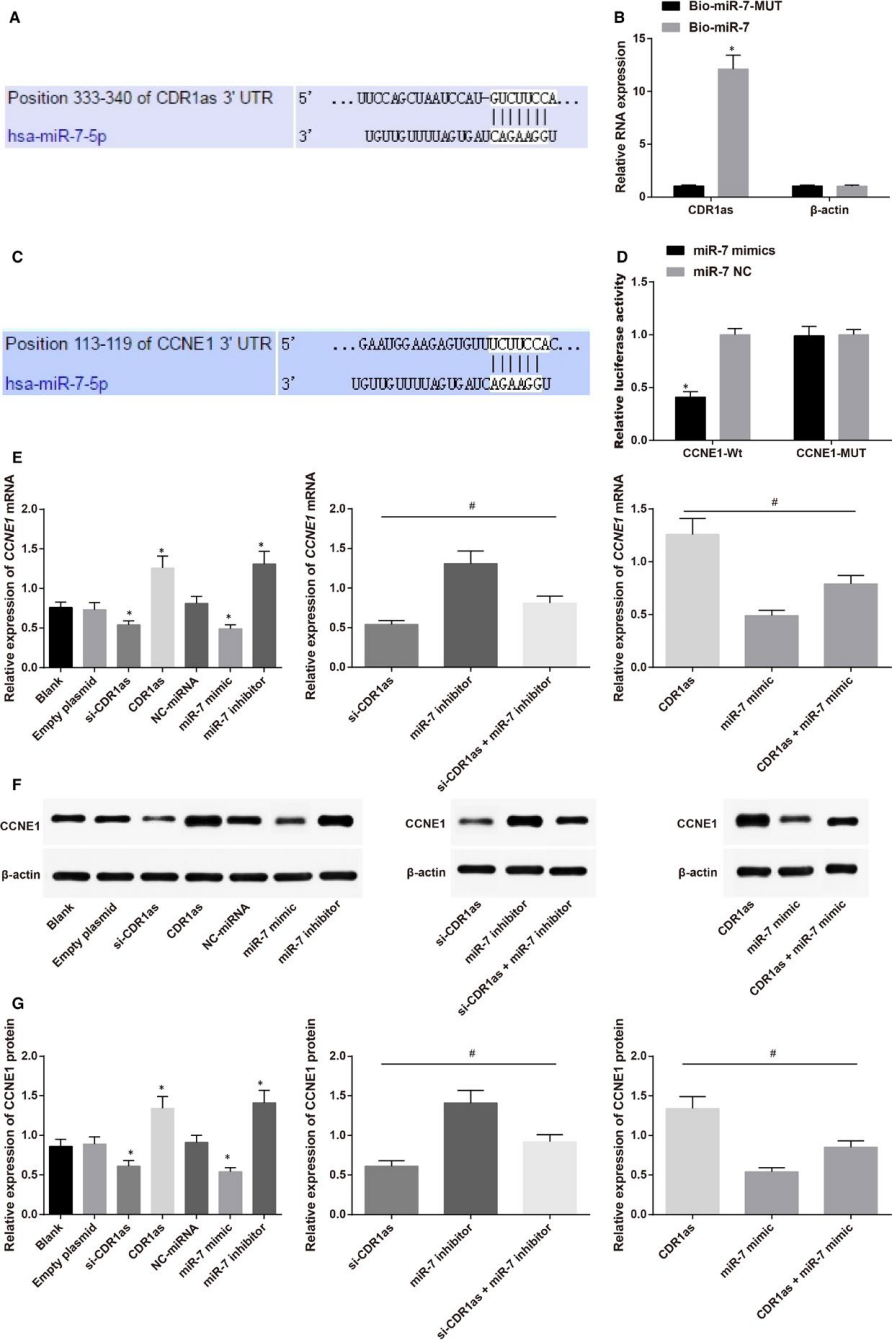
Validation on relationship among CDR1as, miR‐7 and CCNE1 showed that CDR1as competitively inhibits miR‐7 to regulate CCNE1. (A) TargetScan was utilized to predict the bind site of CDR1as and miR‐7; (B), RNA Binding Protein Immunoprecipitation Assay was applied to detect the bind of CDR1as and miR‐7; (C), RT‐PCR was used to determine the expression of CDR1as; (D), TargetScan predicted that CCNE1 was a target gene of miR‐7; (E), Double luciferase reporter assay confirmed that CCNE1 is a target gene of miR‐7; (F), Western blot of CCNE1 on MCF‐7/5‐fluorouracil (5‐FU) and MDA‐MB‐231/5‐FU cells in each group; (G), expression of CCNE1 in MCF‐7/5‐FU and MDA‐MB‐231/5‐FU cells in each group; *, compared with other groups, *P < *0.05; #, comparison among groups, *P < *0.05

Targetscan software indicates the bind site of CDR1as and miR‐7 (Figure 3A). To verify whether CDR1as can directly bind miR‐7, we firstly synthesized biotin labeled miR‐7 and transfected into MCF‐7 cells. Then total RNA which was bind miR‐7 was isolated from cell lysis buffer. RT‐PCR was applied to determine the expression of CDR1as. As indicated in Figure 3B, miR‐7 can gather CDR1as in BC cells, but not β‐actin, which suggested that miR‐7 can specially recognize sequences of CDR1as. Targetscan database was used to evaluate the target genes of miR‐7 and CCNE1 was selected as a target gene for further analysis (Figure 3C). Double luciferase reporter assay (Figure 3D) showed that in wide type, compared with CCNE1‐Wt + miR‐7 NC group, the luciferase activity of CCNE1‐Wt + miR‐7 mimics group was decreased (*P* < 0.05), while in mutant type, the luciferase activity ofCCNE1‐MUT + NC group and group co‐transfected with CCNE1‐MUT and miR‐7 mimics showed no significant difference (*P* > 0.05), indicating that miR‐7 can inhibit CCNE1. RT‐PCR and Western blot were both performed to detect the expression of CCNE1 in both MCF‐7/5‐Fu and MDA‐MB‐231/5‐Fu cells in each group (Figure 3E‐G). Compared with MCF‐7/5‐Fu and MDA‐MB‐231/5‐Fu cells in Blank group, the expressions of CCNE1 in si‐CDR1as group and miR‐7 mimic group were suppressed, while that in CDR1as group and miR‐7 inhibitor group were increased. In contrast to miR‐7 mimic group, the expression of CCNE1 in CDR1as + miR‐7 mimic group was elevated (*P < *0.05). The expression of CCNE1 in si‐CDR1as + miR‐7 inhibitor group was increased in comparison to si‐CDR1as group, indicating that CDR1as can competitively inhibit miR‐7 to regulate CCNE1.

### 3.4 Inhibition of miR‐7 reverses the enhancement on chemosensitivity of 5‐FU‐resistant BC cells caused by CDR1as silencing

**Figure 4:**
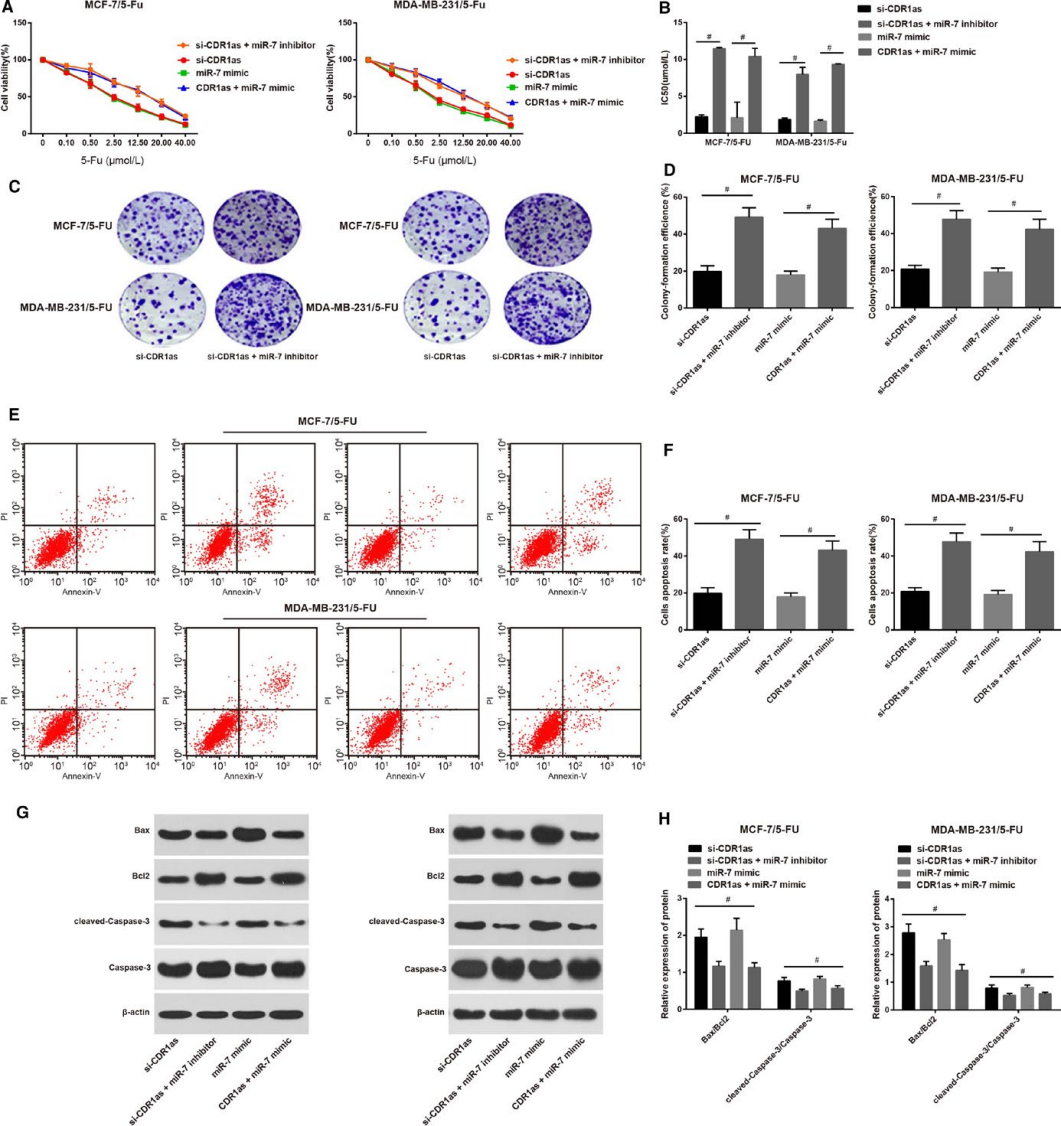
Regulation on miR‐7 and CDR1as can influence the chemosensitivity of 5‐fluorouracil (5‐FU)‐resistant breast cancer (BC) cells. (A) Cell growth curve of 5‐FU‐resistant BC cells; (B), IC50 of 5‐FU‐resistant BC cells; (C), colon formation assay on 5‐FU‐resistant BC cells after regulation on miR‐7 and CDR1as; (D), colon formation rate of 5‐FU‐resistant BC cells; (E), cell apoptosis of 5‐FU‐resistant BC cells as detected by FCM; (F), cell apoptosis rate of 5‐FU‐resistant BC cells; (G), western blot on cell apoptosis related factors in 5‐FU‐resistant BC cells; (H), expressions of cell apoptosis related factors in 5‐FU‐resistant BC cells; #, comparisons among groups, *P < *0.05

Comparison on 5‐FU‐resistant BC cells between si‐CDR1as group and si‐CDR1as + miR‐7 inhibitor group, as well as between miR‐7 mimic group and CDR1as + miR‐7 mimic group showed that cell survival rate in each group was decreased along with the increase concentration of 5‐Fu (Figure 4A). Compared with si‐CDR1as group, IC50 (Figure 4B) and colon formation rate (Figure 4C,D) in si‐CDR1as + miR‐7 inhibitor group were increased, while cell apoptosis rate (Figure 4E,F) and expressions of Bax/Bcl2 and cleaved‐Caspase‐3/Caspase‐3 were decreased (Figure 4G,H). Similarly, IC50 (Figure 4B) and colon formation rate (Figure 4C,D) in CDR1as + miR‐7 mimic group were also elevated, but cell apoptosis rate (Figure 4E,F) and expressions of Bax/Bcl2 and cleaved‐Caspase‐3/Caspase‐3 were decreased (Figure 4G,H) were reduced in comparison to miR‐7 mimic group. Those results replied that inhibition of miR‐7 reverses the enhancement on chemosensitivity of 5‐FU‐resistant BC cells caused by CDR1as silencing and overexpression of CDR1as can also reverse the enhancement on chemosensitivity of 5‐FU‐resistant BC cells caused by overexpression of miR‐7, indicating that CDR1as and miR‐7 are in mutual competition.

### 3.5 Implication of CDR1as in development of BC and its effect on chemosensitivity to 5‐Fu

**Figure 5:**
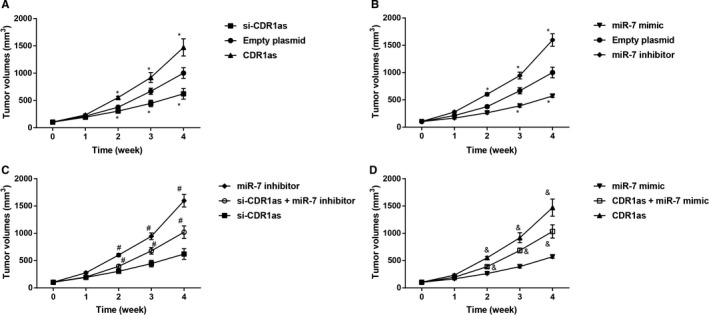
Observation on xenotransplanted tumour size of nude mice in each group. *, compared with Empty plasmid group, *P < *0.05, #, compared with si‐CDR1as group, *P < *0.05, &, compared with miR‐7 mimic group, *P < *0.05

The mice with xenotransplanted tumour were decreased in weight and the tumour sizes were increased. The tumour growth curve illustrated that the tumour growth rate in CDR1as group and miR‐7 inhibitor group was faster than that in Empty plasmid group, while the tumour size in Empty plasmid group was grown in a faster speed than those in si‐CDR1as group and miR‐7 mimic group (Figure 5A,B). Mice in si‐CDR1as + miR‐7 inhibitor group had larger tumour size when compared with si‐CDR1as group at the same time point and the tumour growth speed in si‐CDR1as + miR‐7 inhibitor group was faster than that in si‐CDR1as group (Figure 5C). Those observations demonstrated that silencing of CDR1as in vivo can increase the chemosensitivity of BC resistant cells to 5‐Fu and inhibiting on miR‐7 can reverse the effect on chemosensitivity of 5‐FU‐resistant BC cells caused by silence of CDR1as. The tumour size of CDR1as + miR‐7 mimic group was enlarged when compared with miR‐7 mimic group in the same time point (Figure 5D), indicating that overexpression of CDR1as can also reverse the enhancement on chemosensitivity of 5‐FU‐resistant BC cells caused by overexpression of miR‐7.

## 4 DISCUSSION

Widespread and substantial studies have been focused on the implication of circular RNA on cancer progression.^17^, ^18^, ^19^ CDR1as, one of the well‐known circular RNA, has been suggested to have certain role in certain diseases, including hepatocellular carcinoma.^13^ However, less study was found on the involvement of CDR1as in chemosenstivity of BC.

In this study, we firstly cultivated 5‐FU resistant BC cells and measured the expression of CDR1as and miR‐7 in BC cells. The evidence in this study supported that overexpression of CDR1as can also reverse the enhancement on chemosensitivity of 5‐FU‐resistant BC cells caused by overexpression of miR‐7 and CDR1as may regulate chemosensitivity of 5‐FU‐resistant BC cells by inhibiting miR‐7 to regulate CCNE1. Given the fact that miR‐7 is one of the target genes of CDR1as as evident in previous studies,^20^, ^21^ we used DNA pull down, Luciferase Reporter Assay and online software to predict and verify the relationship of CDR1as and miR‐7. In addition, we further found that miR‐7 can target CCNE1. To learn whether overexpression or inhibition of CDR1as/miR‐7 can affect the cell growth in 5‐FU‐resistant BC cells, we measured the IC50, colon formation rate and cell apoptosis rate in cells. Based on the findings on cell proliferation and cell apoptosis, it is implied that inhibition of CDR1as can increase the chemosensitivity of 5‐FU‐resistant BC cells and overexpression of miR‐7 may increase chemosensitivity of 5‐FU‐resistant BC cells. CDR1as acts as a strong sponge for miR‐7. Studies on in embryonic zebrafish midbrain showed that overexpression of CDR1as can induce developmental defects and regulate insulin transcription and secretion in islet cells through blocking miR‐7.^14^, ^21^ The mechanism of circRNAs in gene regulation has certain relationship with its function of competing endogenous RNAs or microRNA sponges during biological processes and diseases progression even including carcinogenesis.^22^ CDR1as has long been recognized as a tumour oncogene in various genes^23^ in addition to its role in myocardial infarction which suggested that CDR1as was elevated in mice with myocardial infarction under hypoxic treatment, and overexpression of CDR1as can promote cell apoptosis, which can be reversed by overexpression of miR‐7a.^24^ Furthermore, CDR1as also proved to promote hepatocellular carcinoma cell proliferation and invasion and act as a risk factor of hepatic microvascular invasion in hepatocellular carcinoma.^23^

As one of the highlights of this study, we further explore the possible mechanism of CDR1as in regulating chemosensitivity of 5‐FU‐resistant BC cells. The results supported the implication of miR‐7 and CCNE1. MiR‐7, a putative tumour‐suppressor, regulates the expression of several important drivers in multiple types of cancer.^25^ Consistent with the result of our study, an investigation on hepatocellular carcinoma showed that miR‐7 exerts its tumour suppressive function though the inhibition of oncogene CCNE1 expression.^26^ Since miR‐7 interacts with Cdr1as, we examined whether overexpression of miR‐7 can reverse the enhancement on chemosensitivity of 5‐FU‐resistant BC cells caused by CDR1as silencing, we transfected plasma with overexpression of CDR1as and inhibitor of miR‐7 in 5‐FU‐resistant BC cells. The result showed that inhibition of miR‐7 reverses the enhancement on chemosensitivity of 5‐FU‐resistant BC cells caused by CDR1as silencing, which further provided certain ground for the implication of CDR1as and miR‐7 on chemosensitivity of 5‐FU‐resistant BC cells. To confirm the tumorigenic role of CDR1as in vivo, the xenograft tumour model was established and nude mice were administrated tumour cells that were knocked down or had overexpressed CDR1as and/or miR‐7. Observation on tumour size demonstrated that silencing of CDR1as and overexpression of miR‐7 could slow down the tumour growth, which was consistent with above results in this study. The results of xenograft model also implied that silencing of CDR1as could enhance the chemosensitivity of 5‐FU‐resistant BC cells.

## 5 CONCLUSION

Taken together, our results demonstrated a possible mechanism of CDR1as implicating in chemosensitivity of 5‐FU‐resistant BC cells and also provided a new sight into circular RNA network in BC cells. Inhibition of circular RNA CDR1as increases chemosensitivity of 5‐FU‐resistant BC cells by up‐regulating miR‐7, suggesting that CDR1as may be a potential tool in determining and optimize the therapeutic strategies of BC chemotherapy. To its end, the exact role of CDR1as in BC tumour cells requires further investigation since this study only proposed a possible mechanism wherein.

## CONFLICTS OF INTEREST

The authors declare no potential conflicts of interest.
